# Lateral Extra-articular Tenodesis Does Not Alter Early Gait Biomechanics After ACL Reconstruction in a Pediatric Population: A Motion Analysis Study

**DOI:** 10.1177/03635465261487281

**Published:** 2026-10-09

**Authors:** Anton Manitiu, David Mazy, Alexia Bayol, Victoria Blouin, Penelope Saunders, Nicola Hagemeister, Marie-Lyne Nault

**Affiliations:** * Faculté de Médecine, Université de Montréal, Montréal, Québec, Canada; † CHU Sainte-Justine, Montréal, Québec, Canada; ‡ Laboratoire d’Innovation Ouverte (LIO), Research Center CHU Sainte-Justine, Montréal, Québec, Canada; § Department of System Engineering, École de Technologie Supérieure, Montréal, Québec, Canada; Investigation performed at CHU Sainte-Justine, Azrieli Research Center, Montréal, Québec, Canada

**Keywords:** anterior cruciate ligament reconstruction, lateral extra-articular tenodesis, pediatrics, gait biomechanics, knee kinematics, motion analysis, rotational instability

## Abstract

**Background:**

Lateral extra-articular tenodesis (LET) is increasingly added to anterior cruciate ligament reconstruction (ACLR) to reduce graft failure and improve rotatory stability. However, concerns remain that LET may overconstrain the lateral compartment, alter gait biomechanics, and contribute to degenerative changes, especially in pediatric patients.

**Hypothesis/Purpose:**

The purpose of this study was to compare 3-dimensional (3D) knee kinematics during gait between pediatric patients who underwent ACLR with LET (ACLR+LET) and those who underwent isolated ACLR (iACLR). It was hypothesized that patients with ACLR+LET would show modified postoperative gait biomechanics compared to those with iACLR, namely decreased tibial internal rotation and increased valgus during the unloading phase.

**Study Design:**

Cohort study; Level of evidence, 3.

**Methods:**

This retrospective study included pediatric patients (≤18 years of age) who underwent ACLR with or without LET between January 2023 and January 2026. All participants underwent standardized gait assessment with knee kinesiography pre- and postoperatively. Primary outcomes were 3D knee kinematic waveforms in the sagittal, frontal, and transverse planes, analyzed using 1-dimensional statistical parametric mapping.

**Results:**

In total, 70 pediatric patients (70 knees; mean age 15.8 ± 1.3 years) were included: 35 in the iACLR group and 35 in the ACLR+LET group. Preoperatively, a small but statistically significant difference in tibial rotation (1.8°) was identified between 34% to 38% (*P* = .032) and 57% to 59% (*P* = .034) of the gait cycle. At approximately 4 months postoperatively (123.3 ± 58.4 days), no significant between-group differences in knee kinematics were detected in any plane of motion (all *P >* .05). The mean postoperative tibial rotation was 1.03°± 4.27° in the iACLR group versus 1.61°± 3.51° in the ACLR+LET group.

**Conclusion:**

In this pediatric cohort, adding LET to ACLR did not significantly alter early 3D gait biomechanics at approximately 4 months postoperatively. These findings support the safety of LET as an adjunct to ACLR in young patients. Longitudinal studies with higher-demand functional assessments are needed to confirm these results beyond the early postoperative period.

Residual rotatory instability remains a recognized concern after anterior cruciate ligament reconstruction (ACLR), particularly in young and high-demand athletes.^25^ Over the past decade, there has been a renewed interest in combining lateral extra-articular procedures with ACLR to reduce graft failure rates and improve anterolateral knee stability in high-risk populations.^6,25^ Among these, one of the most extensively studied procedures is the lateral extra-articular tenodesis (LET), which uses a strip of the iliotibial band as a nonanatomic soft tissue restraint on the lateral compartment, most often via the modified Lemaire or MacIntosh techniques.^4^ While several randomized controlled trials have demonstrated reduced graft rupture and pivot-shift rates with the addition of LET,^14^ concerns persist among some health professionals that LET may overconstrain the lateral compartment, potentially altering physiological knee kinematics and increasing the risk of lateral compartment degeneration over time.^4,8^

Most biomechanical investigations on LET have focused on cadaveric models or intraoperative stability measures, demonstrating reduced tibial internal rotation and decreased graft forces when LET is added to ACLR.^15,16^ However, these controlled settings do not fully replicate dynamic, functional activities such as walking or running. Only a limited number of in vivo studies have evaluated the effect of LET on knee kinematics after ACLR, and these have involved adult populations assessed using dynamic biplane radiography rather than a clinically accessible system.^3,18^ Chiba et al^3^ compared iACLR and ACLR+LET during downhill running and found that LET contributed little to in vivo kinematics, with a possible transient effect on early sagittal plane stability that was no longer present at 12 months. The impact of LET on weightbearing gait biomechanics in the pediatric population is clinically relevant but remains largely unexplored. Pediatric and adolescent patients typically return to high-demand pivoting sport, exhibit greater ligamentous laxity, and sustain markedly higher graft failure and revision rates than adults, making them strong candidates for LET.^10,20,25,27^

Knee kinesiography (KKG) is a validated, clinically accessible 3-dimensional (3D) motion analysis system that quantifies tibiofemoral kinematics during weightbearing gait with a lightweight exoskeleton, avoiding the skin-motion artifacts that limit conventional marker-based analysis.^11,12^ It has been previously shown to reliably detect biomechanical alterations associated with anterior cruciate ligament (ACL) deficiency and reconstruction in adult and pediatric cohorts, supporting its use as a tool to assess dynamic knee function after surgery.^13,22,23^

We aimed to compare postoperative gait biomechanics in pediatric patients undergoing ACLR with LET (ACLR+ LET) versus isolated ACLR (iACLR) using KKG. We hypothesized that the addition of LET would modify postoperative gait kinematics relative to iACLR, specifically a decrease in tibial internal rotation in the transverse plane.

## Methods

### Ethical Approval

This study was approved by the Research Ethics Board of Centre Hospitalier Universitaire (CHU) Sainte-Justine under protocol number 2024-6689. As it was a retrospective analysis of data collected during routine clinical care, written informed consent was obtained from participants ≥18 years of age, and from a parent or legal guardian together with assent from the participant for those <18 years.

### Study Design

We conducted this retrospective cohort study at the orthopaedic department of the CHU Sainte-Justine, a tertiary pediatric referral center, spanning from January 2023 to January 2026.

### Surgical Cohort

All KKG assessments performed at our institution between January 2023 and January 2026 were reviewed. Patients who underwent primary ACLR and completed both preoperative and postoperative KKG assessments were identified and screened for eligibility. Inclusion criteria were (1) patients aged ≤18 years (2) who underwent ACLR with hamstring tendon autograft (semitendinosus with or without gracilis), either in isolation or combined with a LET, and (3) who completed standardized knee KKG both before and after surgery. Exclusion criteria included (1) patients with a prior ACLR, (2) multiligament knee injuries, (3) fractures requiring fixation, or (4) any condition (musculoskeletal or neurological) affecting gait.

### Surgical Procedures

All ACLRs were performed arthroscopically by 2 fellowship-trained pediatric orthopaedic surgeons (including M.-L.N.) at CHU Sainte-Justine, using a hamstring tendon autograft (semitendinosus with or without gracilis). Hamstring tendon autograft is the standard graft for primary ACLR at our institution in this age group, restricting the cohort to a single graft construct. The semitendinosus was harvested first; if the quadrupled graft diameter was <8 mm, the gracilis tendon was also harvested and combined in a total 6-strand construct to achieve a final graft diameter of 8 to 10 mm. All ACLRs were carried out with an inside-out technique using femoral and tibial adjustable-loop cortical buttons (Infinity; ConMed). LET was gradually integrated into practice at our institution starting in mid-2024, in response to recent literature supporting its benefits in reducing graft failure and improving rotational stability, particularly in young patients who are at greater risk of reinjury due to high activity levels and ligamentous laxity.^21,26,27^ Before that time point, all primary ACLRs were performed in isolation. Given that our tertiary pediatric center exclusively treats this high-risk population, LET was systematically added thereafter as a standardized adjunct for all pediatric ACLRs, rather than reserved for selected cases based on clinical laxity or other preoperative parameters. Aside from the addition of LET from mid-2024 onward, the surgical team, graft choice, fixation method, and the institutional postoperative rehabilitation protocol remained unchanged throughout the study period, limiting temporal confounding between the 2 cohorts. The LET was performed according to the modified Lemaire technique.^9^ A 1 × 8-cm strip of the posterior portion of the iliotibial band was harvested while preserving its distal attachment to the Gerdy tubercle. After ACL fixation, the strip was passed beneath the lateral collateral ligament and fixed to the femur with a suture anchor (TruShot; ConMed) at 30° of knee flexion in neutral rotation.

All patients followed the same standardized, criterion-based rehabilitation protocol. Progression was modified by concomitant meniscal treatment: meniscal repair was managed with a period of restricted weightbearing and range of motion, the duration of which was tailored to the type and size of the repair (typically 2-6 weeks), whereas most meniscectomy, rasping, and untreated cases followed the standard progression. The same criteria were applied identically in both cohorts.

### Data Collection

Knee kinematics were assessed using the KneeKG system (Emovi Inc), a clinically accessible 3D motion analysis tool designed for weightbearing functional evaluation. The system uses a lightweight exoskeleton with rigid thigh and shank attachments, reflective markers, and an infrared tracking unit to capture knee motion during walking.^11^ For each participant, femoral, tibial, and sacral markers were positioned according to the manufacturer’s guidelines (Figure 1). A calibration procedure was performed to define anatomic reference frames and joint axes. After calibration, patients walked on a treadmill at a self-selected comfortable speed. Data were recorded over multiple gait cycles, and kinematic waveforms in the 3 planes were generated using the Knee3D software. The mean gait cycle was computed for each patient from the valid recorded cycles and normalized to 100% of the gait cycle for analysis. All eligible patients with ACLR completed knee KKG both preoperatively and postoperatively as part of their clinical evaluation. Postoperative KKG was performed as part of routine clinical follow-up after surgery and once the patient demonstrated pain-free gait and was cleared by the treating physical therapist. The postoperative assessment occurred at a mean of 123.3 ± 58.4 days (range, 88-285 days); this range reflects clinical scheduling rather than a fixed research protocol.

**Figure 1.**
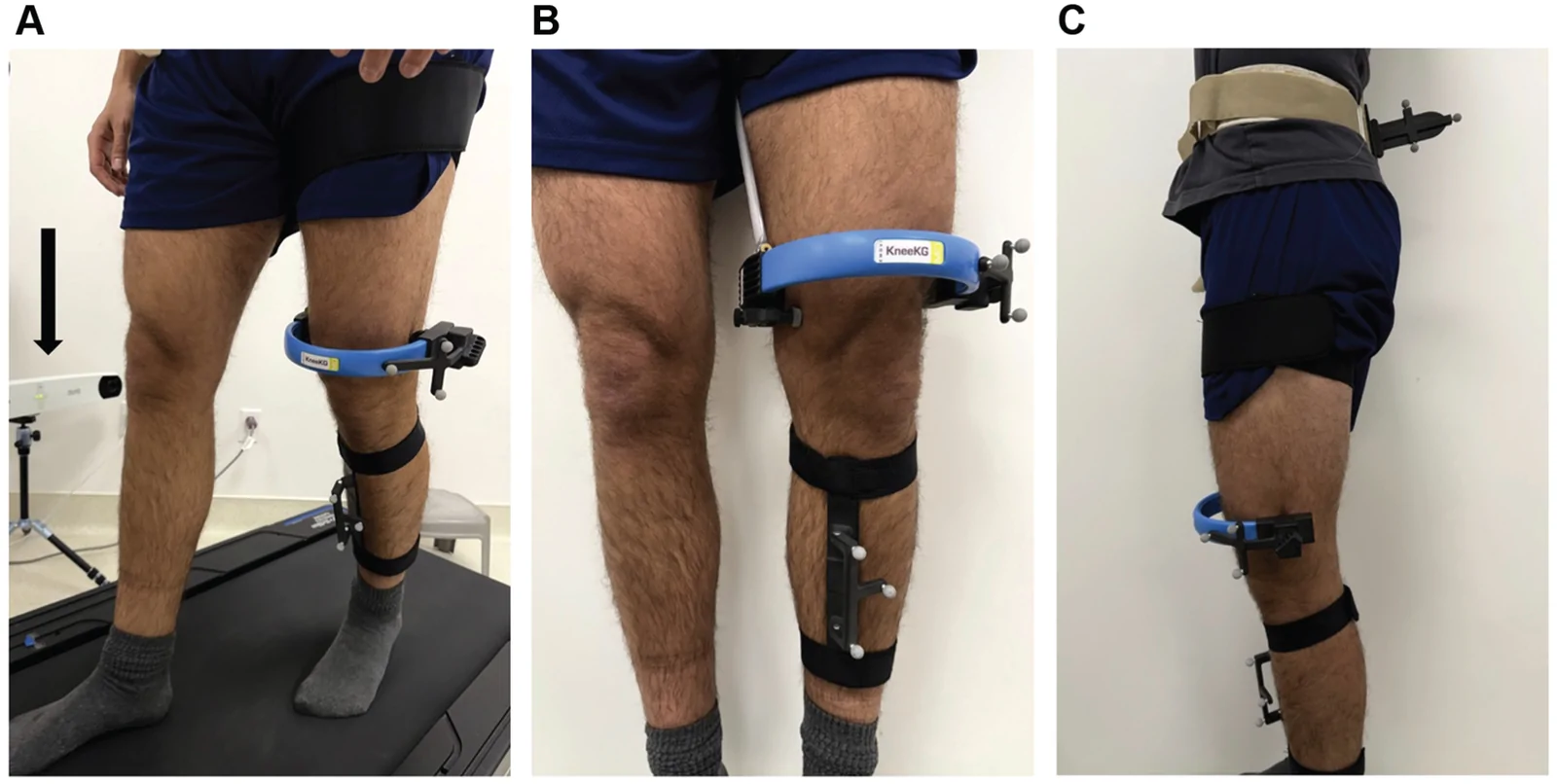
KneeKG system setup for 3-dimensional kinematic gait analysis. (A) Anterior view of a participant standing on a treadmill equipped with the KneeKG system, with the infrared tracking camera (NDI) visible in the background (arrow). (B) Frontal view of tibial and femoral marker placement. (C) Lateral view of a participant walking with the full KneeKG harness, including tibial, femoral, and sacral markers.

### Outcomes Measured

The primary outcomes were 3D knee kinematic waveforms in the sagittal, frontal, and transverse planes throughout the gait cycle. Specifically, flexion/extension, anteroposterior translation, abduction/adduction, and internal/external tibial rotation patterns were analyzed. Additional data collected included spatiotemporal parameters (comfortable walking speed), descriptive characteristics (age, sex, and body mass index [BMI]), and surgical metrics (time from injury to preoperative assessment, time from surgery to postoperative assessment, and meniscal injuries/repair).

### Bias

To minimize selection bias, all consecutive eligible patients meeting inclusion criteria during the study period were included. Because LET was adopted institutionally as a standardized adjunct rather than selected based on clinical laxity, selection bias toward patients with higher instability in the ACLR+LET group was minimized. Assessment bias was reduced by using a single standardized motion analysis system (KneeKG) operated by the same physical therapy technician (P.S.) throughout the study period. One-dimensional statistical parametric mapping (SPM1D) waveform analysis was performed blinded to surgical group allocation.

### Statistical Analysis

Continuous descriptive variables are summarized as means ± standard deviations and were compared between groups using Mann-Whitney *U* tests. Descriptive and categorical comparisons were performed using IBM SPSS Statistics (Version 29.0; IBM Corp). Categorical variables, including sex distribution, were compared using chi-square or Fisher exact tests as appropriate. Kinematic waveform data were analyzed using SPM1D in MATLAB (MathWorks). This approach allows for the comparison of continuous biomechanical waveforms across the gait cycle without reducing data to discrete points. Separate SPM1D *t* tests were performed for each comparison described above, with statistical significance set at an α value of .05. For SPM1D analyses, when no statistically significant difference was detected, the algorithm did not return an exact *P* value, as SPM1D inference is based on cluster-level statistics derived from random field theory rather than point-by-point comparisons^19,20^; in such cases, results are reported as nonsignificant. All analyses were 2-tailed. No imputation was performed for missing data; only patients with complete preoperative and postoperative KKG data sets were included, as specified in the inclusion criteria.

## Results

### Population

Of the 355 patients screened, 70 pediatric patients (70 knees) who underwent ACLR were included, of whom 35 received an additional LET and 35 underwent iACLR. The study flow diagram is presented in Figure 2. All patients who underwent surgery completed both preoperative and postoperative KKG assessments. Descriptive and clinical characteristics for each group are presented in Table 1. There were no statistically significant differences between the 2 groups in terms of age, sex distribution (χ^2^ = 1.59; *P* = .21), BMI, or walking speed. Concomitant meniscal injuries were as frequent in both surgical groups and were recorded by laterality (medial, lateral, or bilateral). Concomitant meniscal injuries were similarly frequent in both groups and were recorded by both laterality (medial, lateral, or bilateral) and treatment (repair, meniscectomy or rasping, or none), with no significant between-group difference in treatment type (Table 1).

**Figure 2.**
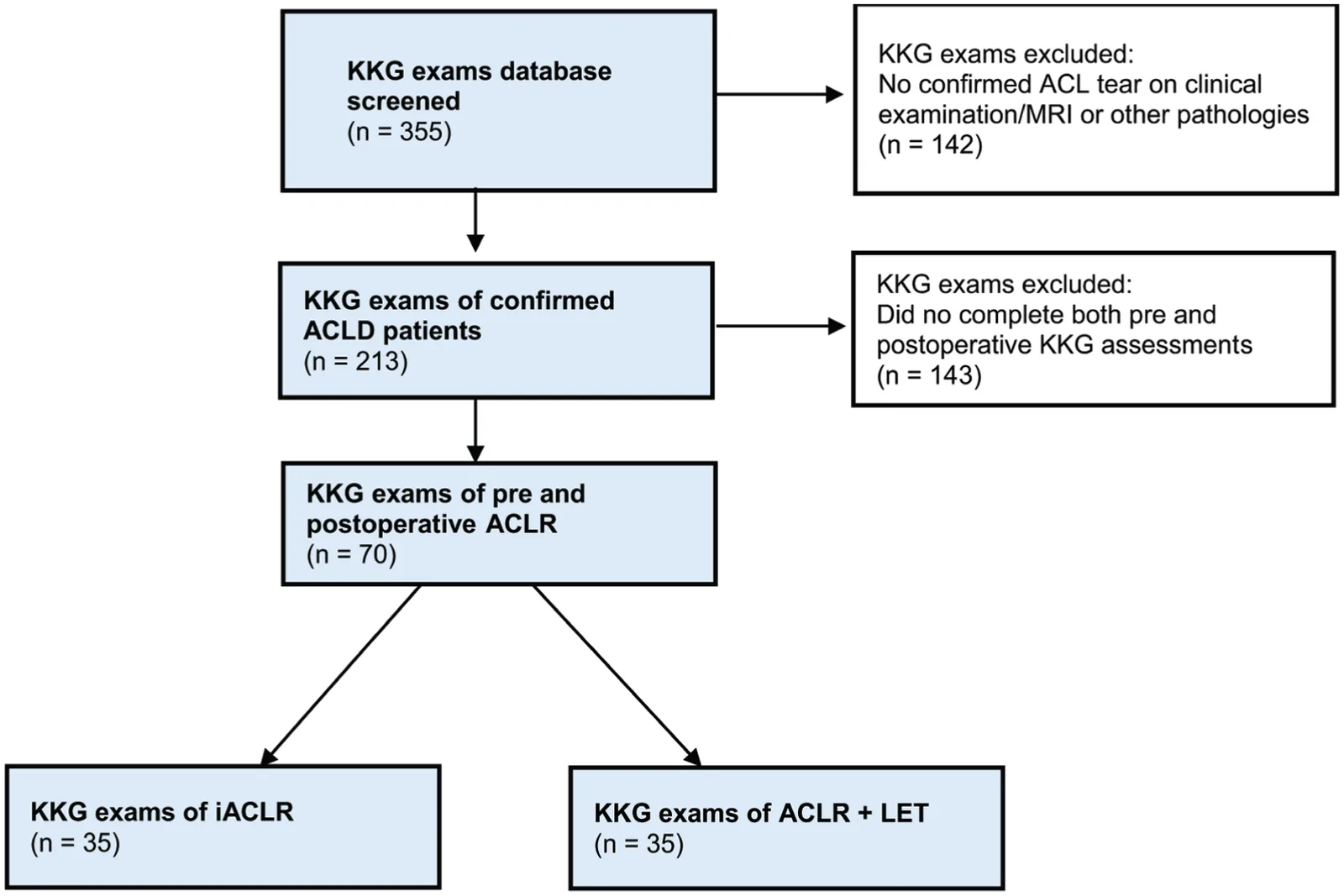
Study participant flow diagram. ACL, anterior cruciate ligament; ACLD, anterior cruciate ligament–deficient knee (preoperative assessment); ACLR, anterior cruciate ligament reconstruction (postoperative assessment); iACLR, isolated anterior cruciate ligament reconstruction; KKG, knee kinesiography; LET, lateral extra-articular tenodesis; MRI, magnetic resonance imaging.

**Table 1:** Characteristics of the Study Participants*^*a*^*

Parameter	iACLR Knees (n = 35)	ACLR+LET Knees (n = 35)	*P* Value
Male/female sex ratio	15/20	9/26	.208
Age, y	16.0 ± 1.3	15.7 ± 1.3	.187
BMI, kg/m^2^	21.3 ± 2.6	21.5 ± 1.9	.762
Preoperative walking speed, mph	2.2 ± 0.4	2.3 ± 0.4	.739
Postoperative walking speed, mph	2.2 ± 0.4	2.2 ± 0.4	.887
Delay trauma to preoperative KKG, days	143.4 ± 105.3	131.2 ± 112.4	.534
Delay ACLR to postoperative KKG, days	108.6 ± 29.0	138.4 ± 74.9	.338
Meniscal injury	20 (57)	26 (74)	.208
Medial meniscus injury	3 (9)	4 (11)	>.999
Lateral meniscus injury	12 (34)	18 (51)	.227
Medial and lateral meniscus injury	5 (14)	4 (11)	>.999
Meniscal repair	13 (37)	18 (51)	.336
Meniscectomy or rasping	7 (20)	8 (23)	>.999

a Values are expressed as mean ± SD or n (%). ACLR, anterior cruciate ligament reconstruction; BMI, body mass index; iACLR, isolated anterior cruciate ligament reconstruction; KKG, knee kinesiography; LET, lateral extra-articular tenodesis.

### Preoperative Comparison Between ACLR+LET and iACLR

As illustrated in Figure 3, preoperative knee kinematics were largely similar between groups across all planes. At approximately 4 months after injury in the entire study cohort (123.3 ± 58.4 days), no significant differences were observed in the sagittal plane, frontal plane, or anteroposterior translation. In the transverse plane, we identified statistically significant but small differences in tibial rotation, between 34% and 38% (*P* = .032) and 57% and 59% (*P* = .034) of the gait cycle, with mean values of 0.43°± 3.59° for iACLR and 2.17°± 3.82° for ACLR+LET. Three-dimensional knee kinematic parameters for both groups are summarized in Table 2.

**Figure 3.**
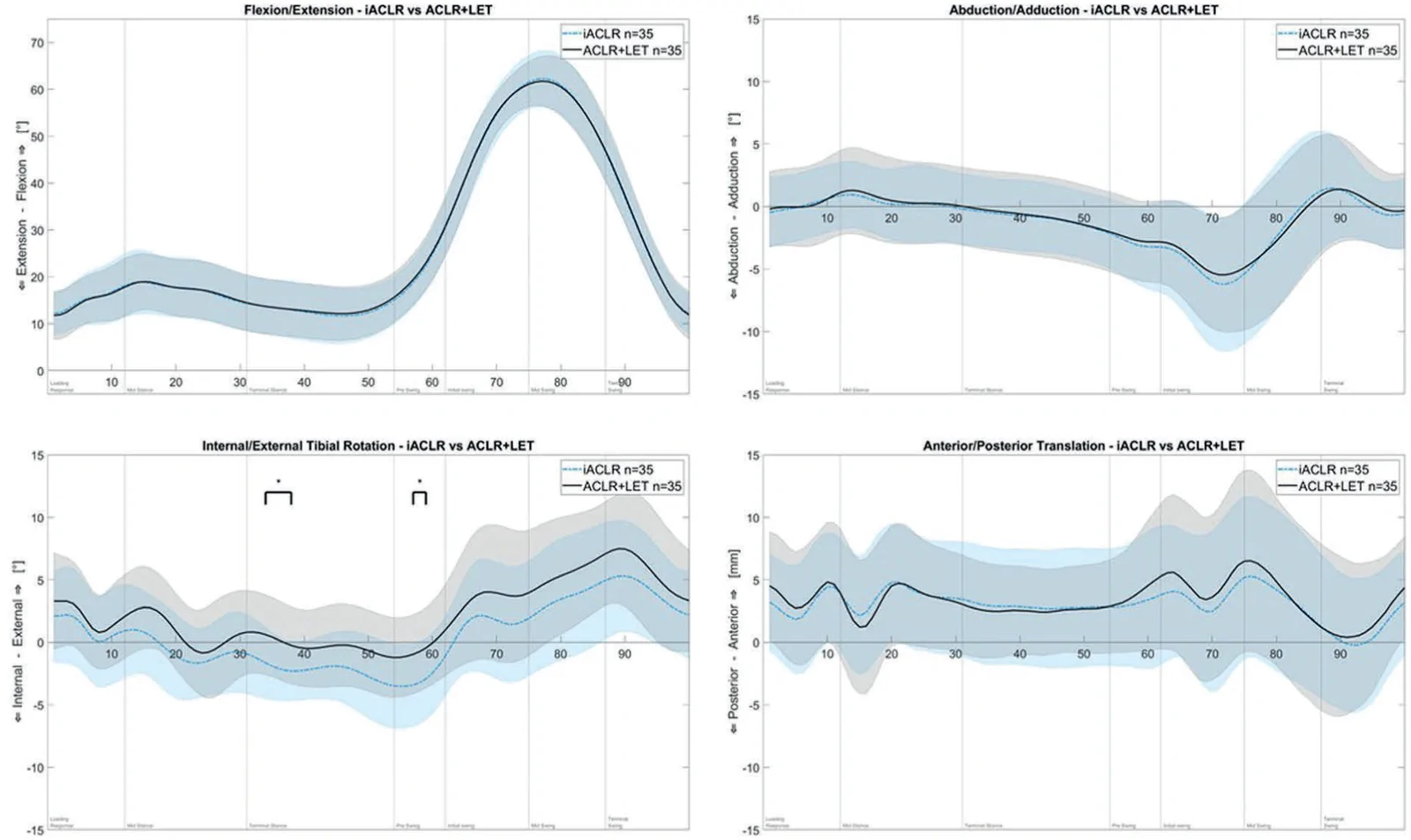
Preoperative knee kinematics during gait: isolated anterior cruciate ligament reconstruction (iACLR) versus anterior cruciate ligament reconstruction (ACLR) + lateral extra-articular tenodesis (LET). Three-dimensional knee kinematic waveforms across the normalized gait cycle (0%-100%) are shown for the sagittal (flexion/extension), frontal (abduction/adduction), transverse (internal/external rotation), and anteroposterior translation planes. Solid lines represent group means; shaded areas represent ±1 SD. *Statistically significant difference identified by 1-dimensional statistical parametric mapping analysis.

**Table 2:** Three-Dimensional Knee Kinematic Parameters During Gait*^*a*^*

	Preoperative			Postoperative		
Kinematic Parameter	iACLR (n = 35)	ACLR+LET (n = 35)	*P* Value	iACLR (n = 35)	ACLR+LET (n = 35)	*P* Value
Flexion/extension						
Mean, deg	14.86 ± 5.84	14.96 ± 5.72	NS	14.45 ± 5.17	13.79 ± 4.83	NS
rom, deg	10.22 ± 3.24	10.04 ± 3.63	NS	9.13 ± 2.25	9.70 ± 2.97	NS
Abduction/adduction						
Mean, deg	−1.21 ± 3.46	−1.03 ± 3.34	NS	−0.93 ± 3.33	−1.90 ± 4.48	NS
ROM, deg	10.26 ± 3.05	9.25 ± 2.50	NS	10.04 ± 3.53	8.71 ± 2.52	NS
Internal/external rotation						
Mean, deg	0.43 ± 3.59	2.17 ± 3.82	.032*^*b*^*	1.03 ± 4.27	1.61 ± 3.51	NS
ROM, deg	11.86 ± 3.92	12.40 ± 3.54	NS	12.97 ± 4.51	12.47 ± 3.34	NS
Anteroposterior translation						
Mean, mm	2.98 ± 5.04	3.31 ± 5.02	NS	2.86 ± 5.25	2.71 ± 4.07	NS
ROM, mm	9.73 ± 3.39	10.56 ± 3.56	NS	10.42 ± 4.38	9.81 ± 3.11	NS

a Values are expressed as mean ± SD. Kinematic waveforms were compared using 1-dimensional statistical parametric mapping (SPM1D) independent-samples *t* tests (α = .05). ACLR, anterior cruciate ligament reconstruction; iACLR, isolated anterior cruciate ligament reconstruction; LET, lateral extra-articular tenodesis; NS, not significant; ROM, range of motion.

b Significant SPM1D clusters at 34% to 38% and 57% to 59% of the gait cycle.

### Postoperative Comparison Between ACLR+LET and iACLR

As illustrated in Figure 4, at approximately 4 months (123.3 ± 58.4 days) postoperative follow-up, 3D gait kinematics remained similar between patients with ACLR+LET and those with iACLR. We observed no significant between-group differences in any plane of motion (all *P* > .05).

**Figure 4.**
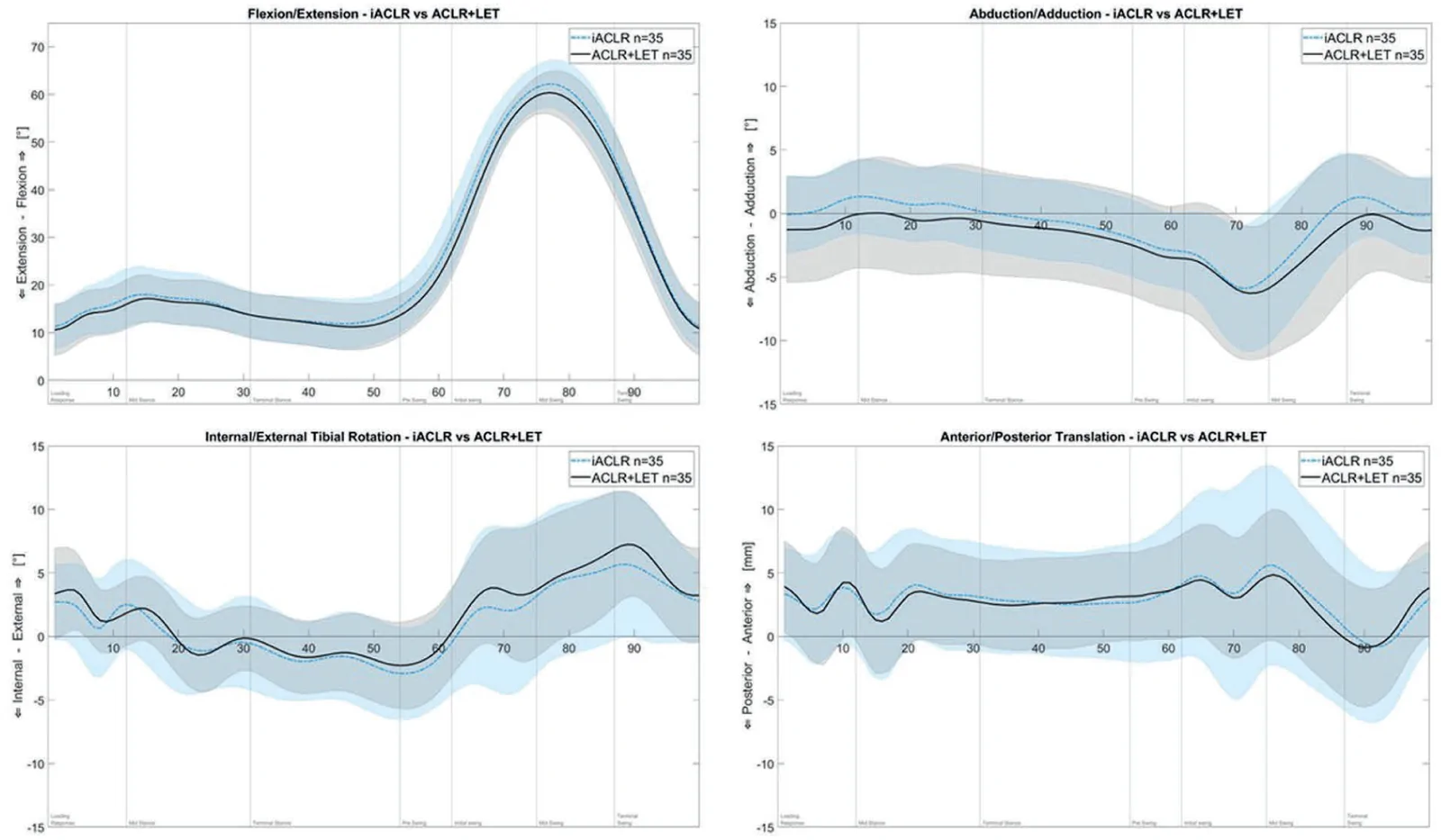
Postoperative knee kinematics during gait: isolated anterior cruciate ligament reconstruction (iACLR) versus anterior cruciate ligament reconstruction (ACLR) + lateral extra-articular tenodesis (LET). Three-dimensional knee kinematic waveforms across the normalized gait cycle (0%-100%) are shown for the sagittal (flexion/extension), frontal (abduction/adduction), transverse (internal/external rotation), and anteroposterior translation planes at approximately 4 months postoperatively. Solid lines represent group means; shaded areas represent ±1 SD. No statistically significant between-group differences were identified by 1-dimensional statistical parametric mapping analysis in any plane.

## Discussion

The most important finding of this study is that adding LET to ACLR did not significantly alter early postoperative gait kinematics during level walking in this pediatric cohort. At approximately 4 months postoperatively, 3D knee kinematics during walking were comparable between the ACLR+LET and iACLR groups across all planes of motion. To our knowledge, this is the first study to evaluate the dynamic effect of LET on gait biomechanics in an exclusively pediatric population using a clinically accessible motion analysis tool. This is clinically relevant because pediatric patients are at particularly high risk of graft failure.^21,27^ The STABILITY randomized controlled trial demonstrated a 67% relative risk reduction in graft failure when LET was added to hamstring ACLR in patients <25 years.^6^ More recently, Kotipalli et al^10^ reported a pooled rerupture rate of only 1.6% after ACLR+LET in pediatric patients, compared with 12.6% after isolated ACLR at a mean follow-up of approximately 40 months. Our findings suggest that these protective benefits found in other studies can be achieved without compromising physiological knee motion during walking.

Small preoperative differences in transverse plane kinematics were observed between groups at 34% to 38% (*P* = .032) of the gait cycle and from 57% to 59% (*P* = .034). Because LET was adopted as a standardized addition to ACLR for the majority of the pediatric patients in our medical center, rather than being selected based on clinical laxity or other kinematic parameter, this difference does not reflect a selection bias toward greater rotational instability in the ACLR+LET group. This difference was confined to 2 brief portions of the gait cycle (4% and 2%) and was small in magnitude (approximately 1.8°). Given this magnitude, the finding falls below both the documented measurement error of the KneeKG system in the transverse plane (2.3°)^11^ and the minimal detectable change values reported for knee rotation in 3D gait analysis (4°-5°),^1,17^ and is therefore unlikely to be clinically relevant. Although the difference also spanned only a brief portion of the gait cycle, no consensus threshold defines the temporal extent a difference must cover to be clinically significant. This interpretation therefore rests on the magnitude of the difference rather than its duration, and the clinical relevance of such small, brief kinematic differences remains to be established and should be interpreted with caution. This result is most consistent with normal intergroup variability between 2 independent temporal cohorts or a type 1 error inherent to waveform-level statistical testing across the full gait cycle. Importantly, this difference was absent postoperatively, and no between-group differences emerged in any plane after surgery. The mean tibial rotation shifted by <1° in each group and in opposite directions, from 0.43° to 1.03° in the iACLR group and from 2.17° to 1.61° in the ACLR+LET group, positive values denoting external rotation. The between-group difference therefore decreased from 1.74° preoperatively to 0.58° postoperatively, and no between-group differences emerged in any plane after surgery.

Several cadaveric studies have shown that LET reduces internal tibial rotation beyond native values,^15,24^ raising the possibility of excessive lateral constraint. However, cadaveric models do not account for neuromuscular adaptation, muscle tone, or proprioceptive feedback that modulate joint kinematics in vivo. Consistent with our findings, Chiba et al^3^ reported minimal kinematic changes after LET in an adult randomized controlled trial at either 6 or 12 months. Their only significant finding was a transient reduction in anterior tibial translation at foot strike at 6 months, which had resolved by 12 months. That LET did not alter tibial rotation even during a high-demand task and at an early time point mirrors our transverse plane findings during early walking and suggests that the absence of rotational effect is consistent across activity levels. Beyond kinematics, Firth et al^5^ found no difference in lateral compartment cartilage quality on quantitative magnetic resonance imaging at 2 years in the STABILITY trial, and Bouchard et al^2^ reported maintained functional outcomes and no accelerated osteoarthritis at a mean follow-up of 15.8 years. Together, these data indicate that LET does not produce clinically meaningful overconstraint.

Several limitations should be acknowledged. First, the 2 surgical groups were created from consecutive temporal cohorts rather than randomly allocated, as LET was adopted institutionally as a standard adjunct to ACLR starting in mid-2024. Although there were no significant differences in baseline characteristics, unmeasured temporal confounders cannot be entirely excluded; a randomized trial comparing ACLR with and without LET in a pediatric population would provide the highest level of evidence. Additionally, gait analysis at 4 months represents an early postoperative time point when neuromuscular recovery is ongoing, and the possibility that LET may progressively influence kinematic patterns with resumption of higher-demand activity and longer-term remodeling of the lateral soft tissue envelope cannot be excluded.^7^ Longitudinal assessments at 6, 12, and 24 months would capture the trajectory of biomechanical recovery and determine whether between-group differences emerge over time, and longer-term follow-up is therefore warranted before definitive conclusions can be drawn regarding the dynamic effect of LET on pediatric knee biomechanics. Walking is also a relatively low-demand activity compared with the cutting and pivoting movements for which LET is primarily indicated; incorporating standardized running gait biomechanics or sport-specific functional tasks such as single-leg hop, lateral cutting, and deceleration maneuvers at these later time points would provide a more complete assessment of the biomechanical impact of LET under conditions that challenge rotational stability. Integrating these assessments within a formal return-to-sport testing framework would further clarify whether biomechanical equivalence during walking translates into functional readiness for pivoting sport. Moreover, only kinematic data were collected; future studies should integrate kinetic measures and electromyographic recordings to detect compensatory neuromuscular strategies that may not manifest as kinematic differences. Concomitant meniscal treatment can modify early rehabilitation, since meniscal repair was generally managed with a period of restricted weightbearing, whereas most meniscectomy, rasping, and untreated cases followed the standard progression. This is a potential source of confounding; however, the distribution of meniscal treatment did not differ significantly between groups, and identical rehabilitation criteria were applied in both cohorts, making differential bias unlikely. Tear pattern was not characterized, and future studies should stratify by this variable to clarify its potential modulatory effect on post-ACLR gait biomechanics.

These findings are most applicable to adolescent patients undergoing primary ACLR with hamstring tendon autograft combined with LET (modified Lemaire). The results may not be generalizable to adult populations, alternative graft choices, and other anterolateral procedures. Furthermore, gait biomechanics during low-demand walking may not reflect kinematic behavior during higher-demand sport-specific activities.

## Conclusion

In this pediatric cohort, adding LET to ACLR did not significantly alter early 3D gait biomechanics at approximately 4 months postoperatively. These findings support the safety of LET as an adjunct to ACLR in young patients. Longitudinal studies with higher-demand functional assessments are needed to confirm these results beyond the early postoperative period.
